# Validation of the Levenson Self-Report Psychopathy Scale in Bulgarian Substance-Dependent Individuals

**DOI:** 10.3389/fpsyg.2020.01110

**Published:** 2020-06-09

**Authors:** Elena Psederska, Georgi P. Yankov, Kiril Bozgunov, Vencislav Popov, Georgi Vasilev, Jasmin Vassileva

**Affiliations:** ^1^Bulgarian Addictions Institute, Sofia, Bulgaria; ^2^Department of Cognitive Science and Psychology, New Bulgarian University, Sofia, Bulgaria; ^3^Department of Psychology, Bowling Green State University, Bowling Green, OH, United States; ^4^Department of Psychology, Carnegie Mellon University, Pittsburgh, PA, United States; ^5^Institute for Drug and Alcohol Studies, Virginia Commonwealth University, Richmond, VA, United States; ^6^Department of Psychiatry, Virginia Commonwealth University, Richmond, VA, United States

**Keywords:** psychopathy, substance use disorders, assessment, adaptation, Bulgaria

## Abstract

The co-occurrence of psychopathy and substance use disorders (SUDs) is associated with higher relapse rates and increased risk of violent offending. Studies on the validity of psychopathy measures in community samples and substance-dependent individuals (SDIs) are scarce. The aim of the current study was to examine the psychometric properties of the Levenson Self-Report Psychopathy Scale (LSRP) in a sample of Bulgarian SDIs and non-dependent controls. We tested 615 participants: 106 heroin users, 91 amphetamine users, 123 polysubstance users, and 295 controls. Confirmatory factor analyses replicated the tri-factor structure of the LSRP (egocentric, antisocial, callous). The scale demonstrated acceptable reliability and validity. SDIs scored significantly higher than controls on the total scale and subscales of the LSRP, indicating good discriminant validity. Overall, results indicate that the LSRP is a valid instrument for measuring psychopathy in Bulgarian community samples.

## Introduction

Psychopathy is considered an extreme variant of antisocial personality disorder (ASPD), consisting of a constellation of affective (e.g., shallow affect, callousness, lack of empathy, lack of remorse), interpersonal (e.g., manipulativeness, egocentricity), and behavioral (e.g., impulsivity, irresponsibility) characteristics (Hare and Neumann, 2008). Given its close relationship to criminal behavior, psychopathy has been usually studied among criminal offenders and has proven to be among the most valid predictors of recidivism (Salekin et al., 1996; Porter et al., 2001), violence (Hare, 1999; Walsh and Walsh, 2006; Thomson et al., 2019a, b), and poor therapeutic outcome (Rice et al., 1992). Nevertheless, psychopathy is an extreme variation of normal personality dimensions (Poythress and Skeem, 2006) and is distributed continuously in community samples (Lilienfeld et al., 2014; Colins et al., 2016). Therefore, some researchers have argued that the primary focus on incarcerated samples limits the scope of research on psychopathy and restricts it to a highly specific group of criminal psychopaths (Brinkley et al., 2001). Lately, the assessment of psychopathy in community samples is attracting increasing research attention as it enables the investigation of the generalizability of the construct of psychopathy and allows comparisons between different populations (e.g., institutionalized and community samples) that may inform targeted intervention strategies.

Psychopathy and substance use disorders (SUDs) are highly comorbid (Smith and Newman, 1990; Derefinko and Lynam, 2007). Rates of SUDs are consistently higher among psychopathic than among non-psychopathic criminal offenders (Smith and Newman, 1990; Blackburn and Coid, 1998; Rasmussen et al., 1999). Similarly, psychopathy is more prevalent among substance-dependent individuals (SDIs) than among the general population (Rutherford et al., 2000). The comorbidity between psychopathy and SUDs has significant implications for the course and treatment outcome of SUDs. Research shows that problem drug use is much more difficult to treat and is associated with higher attrition and relapse rates, increased lifetime sexual HIV risk behaviors, and elevated risk for violent offending in SDIs with high levels of psychopathy (Smith and Newman, 1990; Alterman et al., 1998; O’Neill et al., 2003; Richards et al., 2003; Wilson and Vassileva, 2016), particularly those with high affective psychopathic traits (Durbeej et al., 2014; Swogger et al., 2016). In addition, psychopathy has been associated with more deficient decision-making in SDIs (Vassileva et al., 2007, 2011), which has been related to post-treatment relapse and failure to maintain abstinence (Bowden-Jones et al., 2005; Passetti et al., 2008; De Wilde et al., 2013). Recent machine-learning studies have identified psychopathy as the highest and only common predictor of dependence on different classes of drugs (heroin, amphetamine, cannabis, nicotine, and alcohol), suggesting that psychopathy may be a key diagnostic marker for SUDs, regardless of drug class (Ahn and Vassileva, 2016; Vassileva et al., 2019).

However, the role of psychopathy in SUDs is still not well understood and has been particularly understudied among community samples and in individuals dependent on different classes of drugs. Although the relationship between psychopathy and SUDs has received some attention in the literature (Smith and Newman, 1990; Vassileva et al., 2007, 2011; Walsh et al., 2007; Psederska et al., 2017, 2018), studies on the applicability and validity of different measures of psychopathy in samples of substance-dependent individuals (SDIs) are scarce. Given the significant predictive utility of psychopathy for SUDs (Ahn and Vassileva, 2016; Vassileva et al., 2019), accurate assessment of psychopathy among SDIs is critical, as it could have significant clinical implications for relapse prevention and interventions aimed to decrease criminal behaviors among SDIs.

The “gold standard” for assessing psychopathy in institutionalized populations is the Psychopathy Checklist-Revised (PCL-R; Hare, 1991, 2003), which uses a semi-structured interview format. In addition to the standard PCL-R, two other PCL versions have been developed: the Psychopathy Checklist-Youth Version (PCL-YV; Forth et al., 2003), which assesses psychopathy among adolescents, and the Psychopathy Checklist: Screening Version (PCL:SV; Hart et al., 1995), designed to assess psychopathy in the general population outside of the prison system. The PCL:SV has been successfully validated in a Bulgarian community sample of SDIs, suggesting that it is an adequate tool for assessing psychopathy among substance dependent individuals in the community (Wilson et al., 2014). Although the PCL-R and its versions are excellent and widely used assessment tools, they have some notable limitations. Their administration is time-consuming (requiring ∼1.5 h) and relies on availability of collateral information and on extensive training of research staff in their administration and scoring, which limits their utility in substance abuse clinics and therapeutic communities.

Alternative self-report measures of psychopathy have been developed to facilitate its assessment in the general population and address some of the limitations of the different versions of the Psychopathy Checklist (Hart et al., 1995; Levenson et al., 1995; Lilienfeld and Andrews, 1996; reviewed in Tsang et al., 2018). One of the most widely used among them is the 26-item Levenson Self-Report Psychopathy Scale (LSRP) (Levenson et al., 1995), initially developed to assess psychopathy in individuals who do not manifest extreme levels of the trait. The LSRP was designed to reflect the classical dual-factor model of psychopathy, which distinguishes between primary and secondary subtypes of the disorder (Karpman, 1941; Blackburn, 1975; Vassileva et al., 2005). Primary psychopathy is characterized by personality traits such as callousness, lack of remorse, and feeling of guilt, which are more strongly related to the affective and interpersonal characteristics of the disorder, whereas secondary psychopathy is associated with an impulsive, irresponsible, and antisocial lifestyle, which reflects the behavioral dimension of psychopathy (Hare, 2003). This distinction is supported by factor analytic studies of the original Psychopathy Checklist (PCL; Hare, 1980) and its revised version PCL-R (Hare, 1991).

Although the LSRP was designed to measure psychopathy in the general population, its psychometric properties have been examined primarily in samples of criminal offenders and college students (Levenson et al., 1995; Brinkley et al., 2001, 2008; Sellbom, 2011; Salekin et al., 2014; Shou et al., 2017; Wang et al., 2018), with only few existing studies with community volunteers (Somma et al., 2014; Popov et al., 2015; Garofalo et al., 2018). Psychometric studies of the LSRP report acceptable internal consistency and adequate convergent and discriminant validity (Brinkley et al., 2001, 2008; Sellbom, 2011; Shou et al., 2017; Garofalo et al., 2018). However, one of the major limitations of the LSRP is that its validity as a diagnostic tool for psychopathy has not been rigorously examined. Most studies examining the external validity of the LSRP have focused on whether the pattern of correlations between the LSRP and various personality traits is similar to the one demonstrated by studies with the PCL-R (e.g., Levenson et al., 1995; Lynam et al., 1999; Brinkley et al., 2008; Miller et al., 2008; Sellbom, 2011; Somma et al., 2014). The few studies that have directly compared the LSRP and the PCL-R have questioned the validity of the LSRP as a measure of psychopathy based on the significant but medium-size correlations (*r* = 0.30–0.35) found between their total scores (Brinkley et al., 2001; Poythress et al., 2010).

With regards to its factor structure, investigations have found support for different factor solutions of the LSRP across samples. Lynam et al. (1999) used confirmatory factor analysis and replicated the original dual-factor model of the LSRP. More recently, Brinkley et al. (2008) extracted a different factor structure through an exploratory factor analysis, which identified three factors – *egocentric*, *callous*, and *antisocial*. The 3-factor structure of the LSRP is the most widely accepted in the literature and has been successfully replicated by Salekin et al. (2014) in the United States; by Sellbom (2011) in England; by Somma et al. (2014) in Italy; by Garofalo et al. (2018) in the Netherlands; and by Shou et al. (2017) and Wang et al. (2018) in China. Our research team has evaluated the psychometric properties of the LSRP in a Bulgarian sample, including a subset of participants in the current study (Popov et al., 2015), which, to our knowledge, is the only study with the LSRP in SDIs. We identified a four-factor structure of the LSRP (*deceitful/manipulative*, *superficial/selfish*, *callous*, and *antisocial*), which closely resembled the four-facet structure of the PCL-R, extracted by Hare (2003). Our previous findings suggest that the LSRP is a valid measure of psychopathy in SDIs that can be used as a screening tool prior to conducting the more time- and resource-consuming PCL interviews (Popov et al., 2015).

### Objectives of the Study

Our study has four main goals. First, we build upon our previous study with the LSRP in Bulgaria (Popov et al., 2015) and expand our knowledge of the applicability of the LSRP to different subtypes of substance-dependent populations. A second goal is to establish the reliability of the LSRP in Bulgaria. To this end, in addition to the original 2-factor and the alternative 3-factor models of the LSRP, we also test the previously identified 4-factor solution (Popov et al., 2015), referred to as the “experimental” model. We also conduct measurement invariance analyses on the best-fitting factor solution to verify that the LSRP can be used with the same measurement properties in substance-dependent populations as in the general population. Third, we assess the LSRP’s construct validity and examine if it measures the same construct as the more time- and labor-intensive PCL:SV. Finally, we examine potential gender differences and group differences in psychopathy in individuals dependent on different classes of drugs [heroin-dependent individuals (HDIs), amphetamine-dependent individuals (ADIs), and polysubstance-dependent individuals (PDIs)], and the patterns of associations between psychopathy and theoretically related external variables. Based on the majority of studies of the psychometric characteristics of the LSRP (Brinkley et al., 2008; Sellbom, 2011; Salekin et al., 2014; Somma et al., 2014; Shou et al., 2017; Garofalo et al., 2018; Wang et al., 2018), we expect to find three factors in our Bulgarian sample, which will be correlated with theoretically related variables and will distinguish between substance-dependent and non-dependent groups. We also hypothesize that HDIs, ADIs, and PDIs will score significantly higher on the LSRP than non-substance-dependent participants.

## Materials and Methods

### Participants

Participants were recruited from a larger ongoing study on impulsivity among substance-dependent individuals in Bulgaria via flyers placed at substance abuse clinics and therapeutic communities, as well as through the study’s web page and Facebook page. Participants were initially screened via telephone on their medical and substance use histories. All participants had to meet the following inclusion criteria: (1) age between 18 and 50 years, (2) Raven’s Progressive Matrices (Raven, 2000) estimated IQ higher than 75, (3) minimum of 8th grade education, (4) being able to read and write in Bulgarian, (5) HIV-seronegative status, and (6) negative breathalyzer test for alcohol and negative urine toxicology screen for amphetamines, methamphetamines, cocaine, opiates, methadone, cannabis, benzodiazepines, barbiturates, and MDMA. Exclusion criteria included history of neurological illness, head injury with loss of consciousness of more than 30 min, and history of psychotic disorders and/or use of antipsychotic medication.

Participants included 615 individuals (402 males and 213 females), with a mean age of 28.2 years (*SD* = 6.9). From those, 106 participants had a history of heroin dependence (79 males, 27 females), 91 had a history of amphetamine dependence (57 males, 34 females), and 123 had a history of polysubstance dependence (101 males, 22 females). The control group (*N* = 295; 165 males, 130 females) included 203 participants (125 males, 78 females) with no past or current history of abuse or dependence on any substance, 54 non-substance-dependent siblings of heroin users (24 males, 30 females), and 38 non-substance-dependent siblings of amphetamine users (16 males, 22 females). The majority of participants with a history of substance dependence were in protracted abstinence at the time of testing (i.e., full sustained remission for more than 1 year by DSM-IV criteria) (American Psychiatric Association, 2000) – on average 6.74 (*SD* = 5.79) years for the heroin group, 3.28 (*SD* = 2.97) years for the amphetamine group, and 2.96 (*SD* = 3.71) years for the polysubstance group. Please see Table 1 for participants’ characteristics.

**TABLE 1:** Descriptive statistics and group differences in demographic variables and measures of psychopathy.

	Controls (C)	HDIs (H)	ADIs (A)	PDIs (P)	*p*	Contrast
N	295	106	91	123		
Age	28.00 (7.54)	31.89 (5.81)	25.40 (5.67)	27.54 (5.95)	0.001	H > C, A, P C > A
Years of education	14.40 (2.76)	12.83 (2.51)	13.28 (2.34)	13.16 (2.46)	0.001	C > H,A,P
Estimated IQ	109.07 (14.22)	104.86 (12.69)	108.8 (11.88)	107.28 (13.26)	0.042	C > H
Years drug use	−	7.03 (3.24)	4.22 (2.96)	6.22 (4.28)	0.001	H,P > A
Length of abstinence	−	6.74 (5.79)	3.28 (2.97)	2.96 (3.71)	0.001	H > A,P
PCL:SV F1	1.6 (1.77)	5.01 (2.68)	3.38 (2.64)	5.06 (2.82)	0.001	C < H,A,P H,P > A
PCL:SV F2	1.83 (2.18)	7.12 (2.75)	5.63 (2.81)	7.89 (2.69)	0.001	C < H,A,P H,P > A
PCL:SV Total	3.43 (3.48)	12.15 (4.86)	9.00 (4.83)	12.94 (4.85)	0.001	C < H,A,P H,P > A
LSRP F1	12.89 (7.22)	16.60 (7.92)	15.36 (8.29)	17.23 (7.50)	0.001	C < A,H,P
LSRP F2	9.13 (4.51)	11.66 (4.83)	11.22 (4.18)	12.77 (4.37)	0.001	C < A,H,P
LSRP total	22.02 (9.65)	28.26 (11.14)	26.58 (10.60)	30.00 (9.78)	0.001	C < A,H,P

**HDIs, heroin dependent individuals; ADIs, amphetamine dependent individuals; PDIs, polysubstance dependent individuals; C, Controls; H, heroin dependent individuals; A, amphetamine dependent individuals; P, polysubstance dependent individuals; PCL:SV F1, Psychopathy Checklist: Screening Version Factor 1; PCL:SV F2, Psychopathy Checklist: Screening Version Factor 2; PCL:SV Total, Psychopathy Checklist: Screening Version Total Score; LSRP F1, Levenson Self-Report Psychopathy Scale Factor 1; LSRP F2, Levenson Self-Report Psychopathy Scale Factor 2; LSRP Total, Levenson Self-Report Psychopathy Scale Total Score.**

### Procedures

The study was approved by the Institutional Review Boards of Virginia Commonwealth University and the Medical University in Sofia on behalf of the Bulgarian Addictions Institute. Subjects who met inclusion criteria were contacted via telephone and invited to participate in the study. All participants gave written informed consent. Abstinence from alcohol and drug use at the time of testing was verified by Breathalyzer test (Alcoscan AL7000) and urine toxicology screen for amphetamines, barbiturates, benzodiazepines, cannabis, cocaine, MDMA, methadone, methamphetamines, and opiates. All participants were HIV-seronegative, determined by rapid HIV testing.

Testing was conducted by an experienced team of trained psychologists at the Bulgarian Addictions Institute, Sofia, Bulgaria. Data were collected in two sessions of approximately 4 h each, conducted on two separate days. The assessment battery included a combination of clinical interviews, self-report questionnaires, and computer-based neurobehavioral tests. The first session included assessment of SUDs, externalizing psychopathology (e.g., psychopathy, ASPD), and intelligence. The second session included completion of neurocognitive tasks and self-report measures of externalizing and internalizing personality traits and disorders (e.g., depression, alexithymia). Participants were paid a total of 80 Bulgarian leva (approximately 50 USD) for participation in the study.

### Measures

Some of the self-report measures (i.e., Levenson Self-Report Psychopathy Scale, Psychopathy Checklist: Screening Version, Wender Utah Rating Scale, Toronto Alexithymia Scale-20, Aggression Questionnaire) were translated and validated in Bulgarian by our research team. Other measures (i.e., Beck Depression Inventory-II, State Trait Anxiety Inventory, Sensation Seeking Scale) were unpublished Bulgarian translations of the original instruments that were provided to us by colleagues in Bulgaria and were included in some of our previous publications (Vassileva et al., 2007, 2011, 2019; Ahn et al., 2014; Wilson et al., 2014; Ahn and Vassileva, 2016; Long et al., 2018; Long et al., 2020). The rest of the instruments (Structured Clinical Interview for DSM-IV, Anxiety Sensitivity Scale, Barratt Impulsiveness Scale-11, UPPS Impulsive Behavior Scale) were translated into Bulgarian by the senior author (JV), a clinical neuropsychologist and a native Bulgarian speaker, and then back-translated into English by Bulgarian psychiatrists and psychologists, including co-authors GV and KB.

#### Assessment of Substance Use Disorders

Substance dependence was assessed with the *Structured Clinical Interview for DSM-IV – Substance Abuse Module* (SCID-SAM; First et al., 1996). Participants who met lifetime criteria for amphetamine dependence and had no history of dependence on any other substances were assigned to the “amphetamine” group. Individuals who met criteria for heroin dependence with no history of dependence on other drugs were assigned to the “heroin” group. The “polysubstance” group included participants with a history of dependence on more than one substance. The control group consisted of individuals who had no history of abuse or dependence on any substance.

#### The Levenson Self-Report Psychopathy Scale

The *Levenson Self-Report Psychopathy Scale* (*LSRP*; Levenson et al., 1995) was developed to assess psychopathic traits and behaviors in the general population. The scale includes 26 items graded on a four-point Likert scale (S*trongly Disagree* to *Strongly Agree*). It was developed to reflect the dual-factor model of psychopathy (Hare et al., 1990), with the first 16 items assessing primary psychopathy characterized by emotional deficits and manipulative and selfish behavior, and the remaining 10 items measuring secondary psychopathy, reflecting impulsivity, and antisocial behavior.

#### Measures of Criterion Variables

To establish the construct validity of the LSRP, we used another reliable measure of psychopathy – the *Psychopathy Checklist: Screening Version* (*PCL:SV*; Hart et al., 1995). The PCL:SV consists of a semi-structured interview, which involves the assessment of 12 characteristics of primary and secondary psychopathy on a rating scale of 0 (absent), 1 (somewhat present), and 2 (definitely present). The semi-structured interview for the PCL:SV was conducted by researchers who were initially trained by the senior author, who is the author of the Bulgarian version of the PCL-R with its publisher Multi Health Systems. Additional training and supervision were provided by two of the co-authors, who took part in formal training workshops led by Robert Hare, the author of the PCL instruments. In line with our earlier findings (Wilson et al., 2014), the PCL:SV exhibited good internal consistency for its total score (α = 0.9) and its two factor scores (α = 0.77 and α = 0.86) in the current sample.

The *ASPD module from the Structured Clinical Interview for DSM-IV Axis II Disorders* (*SCID-II*; First et al., 1997) was used to assess Conduct Disorder (CD) and ASPD. The symptoms related to these disorders were scored on a scale of 1 (absent), 2 (subthreshold), and 3 (present), based on behavioral examples given by the participant throughout the interview. The dependent variable in the current study was the number of symptoms scored with a “3.”

The *Wender Utah Rating Scale* (*WURS*; Ward et al., 1993) is a 25-item self-report scale for retrospective assessment of childhood symptoms of attention deficit hyperactivity disorder (ADHD) in adults. Items are rated on a five-point Likert scale (from *Not at all or slightly* to *Very much*). The scale displayed excellent internal consistency in the current sample (α = 0.92), in line with the earlier evaluation of the psychometric properties of the Bulgarian version of the WURS (Nedelchev et al., 2016).

The *Aggression Questionnaire* (*AQ*; Buss and Warren, 2000) is a revision of the Buss–Durkee Hostility Inventory (Buss and Durkee, 1957). The questionnaire consists of 34 items, rated on a five-point Likert scale. We used the recently validated Bulgarian version of the AQ (Popov et al., 2016a), which has a four-factor structure: *physical aggression*, *verbal aggression*, *hostility*, and *anger.* The entire scale exhibited excellent internal consistency in the current sample (α = 0.91).

The *State-Trait Anxiety Inventory* (*STAI*; Spielberger et al., 1983) is a self-report instrument with two sections, each comprised of 20 items. The first section measures situational “state” anxiety, whereas the second one measures anxiety as a relatively stable personality trait (Spielberger, 2010). Answers are scored on a four-point Likert scale. In the present study, we used the existing Bulgarian adaptation of the scale (Shtetinski and Paspalanov, 2007). Both the state and the trait subscales of the STAI showed excellent internal consistency in this sample (α = 0.89 and α = 0.90, respectively).

The *Anxiety Sensitivity Index* (*ASI*; Reiss et al., 1986) measures sensitivity toward the symptoms of anxiety, a.k.a. “fear of fear,” demonstrated to be an independent construct implicated in susceptibility to addiction (Stewart and Kushner, 2001; Castellanos-Ryan and Conrod, 2012). It consists of 16 items, rated on a five-point scale (from *Strongly disagree* to *Strongly agree*). The scale exhibited good internal consistency in the current sample (α = 0.85).

The *Beck Depression Inventory-II* (*BDI-II*; Beck et al., 1996) is a 21-item self-report questionnaire, assessing current symptoms of depression. Participants rate the degree to which they have experienced specific symptoms of depression during the past 2 weeks. The BDI-II is scored on a four-point Likert scale. We used the existing (unpublished) Bulgarian translation of the scale, which had good internal consistency in the current sample (α = 0.86).

The *Toronto Alexithymia Scale-20* (*TAS-20*; Bagby et al., 1994a, b) is a self-report measure of alexithymia, associated with difficulties in identifying, describing, and interpreting emotions (Sifneos, 1973). The scale includes 20 items rated on a five-point Likert scale. We used the recently validated Bulgarian version of the TAS-20 (Popov et al., 2016b), which had good internal consistency in the present sample (α = 0.82).

The *Barratt Impulsiveness Scale – 11th Edition* (*BIS-11*; Patton et al., 1995) is a 30-item self-report questionnaire consisting of three subscales measuring different dimensions of trait impulsivity: attentional, motor, and non-planning impulsivity. Items are rated on a four-point Likert scale. In the current sample, the total scale exhibited good internal consistency (α = 0.84).

The *UPPS-P Impulsive Behavior Scale (UPPS*; Lynam et al., 2006) is a 59-item self-report scale assessing five distinct trait impulsivity dimensions: *(lack of) premeditation (lack of), perseverance*, *sensation seeking*, *negative urgency*, and *positive urgency* (Cyders and Smith, 2007). Items are rated on a four-point scale. In the present sample, the full scale had excellent internal consistency (α = 0.94).

The *Sensation Seeking Scale-V (SSS-V*; Zuckerman, 1994) is a 40-dichotomous-item scale measuring individual differences in predisposition to seek new experiences. High scores on this scale reflect a higher propensity toward sensation seeking. The SSS-V has 4 subscales – *Disinhibition*, *Thrill and Adventure Seeking*, *Experience Seeking*, and *Boredom Susceptibility*. The scale exhibited good internal consistency in the present sample (α = 0.84).

### Data Analyses

Our main goal was to establish the reliability and validity of the Bulgarian version of the LSRP. First, we present descriptive statistics and internal consistency of the LSRP. We then examine the factor structure of the LSRP using confirmatory factor analysis, testing the original Levenson’s 2-factor structure (Levenson et al., 1995), Brinkley’s 3-factor structure (Brinkley et al., 2008), and the 4-factor structure from our previous study (Popov et al., 2015). Third, we conduct measurement invariance analyses on the best-fitting factor structure, which test how well the hypothesized latent structure fit SDIs and controls^1^. Fourth, we assess gender differences and group differences in psychopathy between heroin, amphetamine, polysubstance users, and controls. Finally, we assess LSRP’s construct, convergent, and discriminant validity by zero-order and partial correlations between LSRP scores and instruments measuring externalizing and internalizing traits and behaviors, and point-biserial correlations with gender.

## Results

### Descriptive Statistics

Table 2 provides descriptive statistics for the full LSRP scale, Brinkley’s 3 factors, and Brinkley’s total score across the different groups.

**TABLE 2:** Descriptive statistics for LSRP across models and samples.

Group	Statistic	Full scale	Brinkley egocentric	Brinkley antisocial	Brinkley callous	Brinkley total
Control	Mean	22.02	8.08	4.77	2.52	15.36
	Median	21.00	7.00	4.00	2.00	15.00
	Mode	18.00	8.00	4.00	0.00	10.00
	*SD*	9.65	5.29	3.02	2.62	7.82
HDIs	Mean	28.26	10.42	6.48	3.67	20.57
	Median	28.00	10.00	7.00	3.50	21.00
	Mode	28.00	8.00	7.00	3.00^a^	20.00^a^
	*SD*	11.14	6.03	3.04	2.43	8.98
ADIs	Mean	26.58	9.80	6.57	3.02	19.40
	Median	25.00	9.00	6.00	3.00	18.00
	Mode	16.00^a^	5.00	6.00	4.00	15.00
	*SD*	10.60	5.97	2.99	2.21	8.38
PDIs	Mean	30.00	10.75	7.24	3.58	21.57
	Median	30.00	10.00	7.00	3.00	22.00
	Mode	22.00^a^	10.00	8.00	3.00	18.00^a^
	*SD*	9.78	5.43	3.01	2.40	8.18
All SDIs	Mean	28.45	10.37	6.80	3.45	20.62
	Median	28.00	10.00	7.00	3.00	20.00
	Mode	25.00	9.00^a^	7.00	3.00	25.00
	*SD*	10.54	5.78	3.02	2.37	8.53

**HDIs, heroin dependent individuals, ADIs, amphetamine dependent individuals; PDIs, polysubstance dependent individuals; SDIs, substance dependent individuals.**

### Internal Consistency

Table 3 displays the internal consistency (Cronbach’s alpha, mean item-total correlations (ITCs) and mean inter-item correlations) of the full scale, Brinkley’s model, and Levenson’s original model, included for reference. Levenson’s two subscales had Cronbach’s alphas of 0.79 and 0.63 across controls, 0.81 and 0.59 across SDIs, and 0.81 and 0.64 across the total sample. Brinkley’s model exhibited similar alpha coefficients to Levenson’s original model. The *egocentric* subscale (F1) had Cronbach’s alphas of 0.78 across controls and 0.80 across SDIs and the total sample. The internal consistencies of the *callous* (F3) and *antisocial* (F2) subscales were lower, ranging from poor to acceptable across groups (α = 0.52–0.69). Brinkley’s model had consistently higher mean item total correlations (*r* = 0.31–0.47) and mean inter-item correlations (*r* = 0.20–0.36) compared to Levenson’s model (*r* = 0.27–0.41 and *r* = 0.13–0.21, respectively).

**TABLE 3:** Internal consistency of LSRP across models and samples.

Model	Sample	Raw alphas	Mean ITCs	Mean inter-item correlation
Levenson total score (26 items)	Controls	0.79	0.32	0.13
	SDIs	0.81	0.34	0.14
	Total	0.82	0.35	0.15
Brinkley total score (19 items)	Controls	0.78	0.35	0.16
	SDIs	0.80	0.38	0.17
	Total	0.81	0.38	0.18
Levenson 2-factor model	Controls	0.79/0.63	0.39/0.30	0.19/0.14
	SDIs	0.81/0.59	0.42/0.27	0.21/0.13
	Total	0.81/0.64	0.41/0.30	0.21/0.15
Brinkley 3-factor model	Controls	0.78/0.62/0.69	0.45/0.37/0.47	0.27/0.24/0.36
	SDIs	0.80/0.57/0.52	0.47/0.33/0.31	0.28/0.20/0.22
	Total	0.80/0.63/0.62	0.47/0.38/0.40	0.28/0.25/0.29

SDIs, substance dependent individuals. *The coefficients for the Levenson and Brinkley models contain multiple values because values are presented in the order of the scales in the respective models.

### Factor Structure

We established LSRP’s factor structure with confirmatory factor analyses (CFA) instead of exploratory factor analyses (EFA) because of the substantial empirical and theoretical research (cf. Salekin et al., 2014) pointing to the replicability of Levenson’s two-factor and Brinkley’s three-factor factor solutions. Thus, we used CFA as a hypothesis-driven method (Brown, 2015), expecting to replicate a two- and a three-factor structure for LSRP.

All LSRP items were measured at the interval level, and we checked if they had multivariate normal distribution. As per Finney and DiStefano’s (2013) guidelines, we explored univariate skewness and kurtosis and found that only six items had an absolute skew above 2 and no items had an absolute kurtosis above 7. Given the undesirable nature of the items (i.e., assessing psychopathic tendencies), the skew of these six items is understandable. As a result, for our CFA analyses, we chose the maximum likelihood estimator over the generalized least squares estimator.

First, we fitted Levenson’s original factor solution to the total sample, as well as separately to the two subsamples – SDIs and controls. Table 4 presents the fit statistics of the original and the other models to the three samples. We included several absolute and comparative fit indices, which Hu and Bentler (1999) and Brown (2015) recommended as robust for structural equation modeling – the root mean square error of approximation (RMSEA), its confidence intervals, the standardized root mean square residual (SRMR), the Tucker–Lewis index (TLI), and the comparative fit index (CFI). The RMSEA, RMSEA’s confidence intervals, and the SRMR measure absolute fit and lower values are recommended – less than 0.05 for RMSEA and 0.08 for SRMR (Brown, 2015). Kline (2011) recommended that RMSEA’s upper confidence interval should also be <0.05 to reject the so-called close-yet-failing models. The TLI and CFI compare with the null model (i.e., measure incremental fit), and values above the cutoffs of 0.90 (Byrne, 1994) and ideally 0.95 (Schumacker and Lomax, 2004) are recommended. Additionally, we included two indices favoring parsimony – the Adjusted Goodness of Fit Index (AGFI; MacCallum and Hong, 1997) which, like CFI and TLI, ranges between 0.00 and 1.00 and has a cutoff of 0.90, and the Bayesian Information Criterion (BIC; Raftery, 1995), which is a comparative index penalizing nested models with increasingly larger sample size and, thus, places a very high value on parsimony.

**TABLE 4:** Fit statistics for the baseline CFA models for controls, SDIs, and total sample.

	Models	Chi-square	df	*p*-value	RMSEA	RMSEA CI−	RMSEA CI+	SRMR	TLI	CFI	AGFI	BIC
Controls	Levenson	981.783	298	0.001	0.088	0.082	0.094	0.092	0.517	0.557	0.738	19874.865
	Experimental	381.869	164	0.001	0.067	0.058	0.076	0.071	0.798	0.825	0.849	15209.383
	Brinkley	457.167	149	0.001	0.084	0.075	0.093	0.073	0.696	0.735	0.822	14251.950
	Brinkley Mod.	255.905	145	0.001	0.051	0.041	0.061	0.064	0.888	0.905	0.892	14073.436
SDIs	Levenson	684.039	298	0.001	0.064	0.057	0.070	0.070	0.702	0.727	0.819	22125.969
	Experimental	306.095	164	0.001	0.052	0.043	0.061	0.059	0.857	0.877	0.883	16922.675
	Brinkley	355.658	149	0.001	0.066	0.057	0.075	0.065	0.786	0.813	0.857	16079.188
	Brinkley Mod.	202.078	145	0.001	0.035	0.023	0.046	0.051	0.939	0.948	0.914	15948.669
Total	Levenson	1317.699	298	0.001	0.075	0.071	0.079	0.072	0.640	0.670	0.814	42042.650
	Experimental	507.467	164	0.001	0.058	0.053	0.064	0.057	0.840	0.862	0.895	32176.830
	Brinkley	651.993	149	0.001	0.074	0.068	0.080	0.061	0.758	0.789	0.867	30410.309
	Brinkley Mod.	308.446	145	0.001	0.043	0.036	0.049	0.049	0.919	0.932	0.932	30092.449

As presented in Table 4, all models had significant chi-squares, something common with models with larger sample sizes such as ours, hence the need for additional fit indices to appraise the models’ goodness-of-fit (Brown, 2015). Fitting Levenson’s original two-factor solution of the LSRP produced a model with bad fit with TLI and CFI in the 0.50 s for the controls and 0.70 s for the SDIs. For the experimental four-factor model, fit was substantially better, with TLI and CFI in the upper 0.85 s, and RMSEA close to the 0.05 cutoff. Notably, this model produced better results for the SDIs. Compared to the experimental model, Brinkley’s three-factor model had slightly worse fit – the TLI and CFI returning to the upper 0.70 s and RMSEA’s lower confidence interval above 0.05. Therefore, we explored the modification indices of Brinkley’s model and found four residual covariances with extremely high values – between items 2 and 3, 4 and 5, 12 and 14, and 24 and 25.

Each pair consisted of items which were part of the same latent factor, and their content gives theoretical grounds to suggest that they share a variance beyond the one accounted for by the latent factor itself. For example, items 2 and 3 both assess lack of remorse (“For me, what’s right is whatever I can get away with” and “In today’s world, I feel justified in doing anything I can get away with to succeed”), whereas items 4 and 5 reflect striving for personal gain (“My main purpose in life is getting as many goodies as I can” and “Making a lot of money is my most important goal”). Similarly, items 12 and 14 evaluate callous tendencies, with both items reversed (“I make a point of trying not to hurt others in pursuit of my goals” and “I feel bad if my words or actions cause someone else to feel emotional pain”), and items 24 and 25 assess quick-temperedness (“I have been in a lot of shouting matches with other people” and “When I get frustrated, I often ‘let off steam’ by blowing my top”). Sellbom (2011) also selected two of our four correlated residuals – between item 2 and 3, and items 24 and 25. Using Brinkley et al.’s (2008) categorization, Factor 1 is the *egocentric* factor, factor 2 is the *antisocial* factor, and factor 3 is the *callous* factor.

After modifying the model with these constraints, Brinkley’s factor solution fitted the data well. Its RMSEA’s upper confidence interval was not greater than 0.05 except for the controls (0.06), the CFI and TLI were above 0.90 except for control’s TLI, and it had the highest parsimony-favoring AGFIs and the lowest BIC information indices. Notably, this adjusted model fitted the SDI group and the total sample better than the control group.

Establishing a well-fitting model for the LSRP was critical for conducting subsequent analyses, which concentrated on comparisons of its latent means between controls and SDIs, and exploring its validity with regards to constructs such as anxiety, aggression, and impulsivity. Although Popov et al. (2015) used EFA and found a four-factor model to best fit the data, the study with that model, which we call *experimental* here, included only 379 participants. In addition, measurement invariance analyses were not conducted in Popov et al.’s (2015) study. In the current study, we used a substantially larger sample size and tested our models in parallel for control and SDI participants. Brinkley’s three-factor model performed the best among the original two-factor solution and the experimental four-factor solution, especially in terms of the parsimony of its fit. We slightly modified the model by correlating four pairs of residuals, but these modifications have already been implemented in Sellbom (2011).

### Measurement Invariance Analyses

Conducting measurement invariance (MI; for review see Vandenberg and Lance, 2000) analyses for the LSRP was a required step in the Bulgarian adaptation of the LSRP. A psychometric instrument is invariant when members of different populations who have the same standing on the construct measured by the instrument receive the same score on the instrument (Schmitt and Ali, 2015). In our case, MI is required for the LSRP to guarantee that the instrument will reliably and similarly measure self-reported psychopathy in SDIs and controls. The culture and language of both populations are the same, but it is unknown whether other differences between the two populations (e.g., SDIs’ substance use or impulsivity) might affect individuals’ observed scores and standing on LSRP’s psychopathy latent factors. Furthermore, if such differences exist, they might affect the latent factors’ intercorrelations and their correlations with other latent constructs related to psychopathy.

MI analyses proceed in several steps where invariance of the model parameters is established in successively fitting increasingly restricted models. The exact number of these steps varies (Schmitt and Kuljanin, 2008), but four steps are common for all approaches. In the first *configural* invariance step, it is demonstrated that the two groups to which the model is fitted have the same number of latent factors and similar pattern of factor loadings. In the second step, called *metric* or “strong” invariance (Horn and McArdle, 1992), the factor loadings are constrained to equality across groups. In the third step, called *scalar* invariance, the intercepts of the regression equations of the items (i.e., the indicators of the latent factors) are constrained to equality across groups. The fourth step, also called *strict* invariance, tests for the uniqueness of the residuals of the observed variables (i.e., the items, the indicators of the model). The fifth, non-required, *factor* invariance step tests for the equality of the variances and covariances of the latent factors, addressing the structural part of the model (vs. the measurement part as established in steps 1–4).

In our MI analyses, we fitted the modified three-factor Brinkley model simultaneously to SDI and control participants. Table 5 presents the steps in LSRP’s measurement equivalence analyses. Each model was compared for equivalence with the model following it through ANOVAs on their chi-squares. Also, the fit of each model’s parameters was explored with lavaan’s *lavTestScore* function. Similar to the CFA analyses, lavaan provides various fit indices to compare the fit of the models. Notably, the comparative fit index Akaike Information Criterion (AIC) has a similar interpretation as BIC, but does not penalize for more complex models. The models with highest TLI and CFI, lowest RMSEA, and lowest AIC and BIC are considered optimal. Cheung and Rensvold’s (2002) simulation study recommends using CFI changes of 0.01 to distinguish significant changes in fit.

**TABLE 5:** Steps in LSRP’s measurement invariance analyses.

Invariance models	Chi-square	df	RMSEA	TLI	CFI	AIC	BIC
Configural	457.983	290	0.043	0.913	0.926	29762.758	30328.517
Metric	471.400	306	0.042	0.919	0.927	29744.175	30239.214
Scalar	560.149	322	0.049	0.889	0.895	29800.924	30225.243
Scalar – intercepts for items 11, 13, 15, 16, 17, and 24 freed	482.647	316	0.041	0.921	0.927	29735.421	30186.261
Strict	539.474	335	0.045	0.908	0.910	29754.248	30121.108
Strict – residuals for items 2, 6, 7, 13, and 15 freed	498.759	330	0.041	0.923	0.926	29723.534	30112.494
Factor	524.742	336	0.043	0.915	0.917	29737.517	30099.956
Factor – variance of factor 3 freed	509.035	335	0.041	0.922	0.923	29723.810	30090.670

First, the configural model was Brinkley’s modified model, which we fitted to both groups. There was no significance testing for this first step as the configural invariance was established in the previous section. Second, the item loadings were constrained to equality in both groups, which did not result in significant change with the configural model [χ^2^_*diff*_(16) = 13.4, *p* = 0.64]. Third, constraining intercepts to equality (scalar invariance) produced misfit with the metric model [χ^2^_*diff*_ (16) = 88.75, *p* < 0.001]. Items 11, 13, 15, 16, 17, and 24 had very high values on the multivariate score test. That is, the omnibus Lagrange multiplier test for all equality constraints across the two groups was significant [χ^2^(35) = 100.5, *p* < 0.001], as well as the univariate chi-square tests for these items’ equality constraints (chi-squares of 7.14, 5.38, 12.49, 17.07, 13.70, and 38.79 with 1 df and all *p*’s < 0.01). We freed the intercepts of the six items and compared this model against the strict invariance model where all residuals were constrained to equality across both groups. The comparison was significant [χ^2^_*diff*_ (19) = 56.83, *p* < 0.001]. The Lagrange multiplier test was significant [χ^2^(48) = 79.75, *p* < 0.01], as well as the univariate chi-square tests for the residuals of items 2, 6, 7, 13, and 15 (chi-squares of 4.26, 5.62, 4.97, 18.11, and 6.97, with 1 *df* and all *p*’s < 0.05). After freeing these residuals, the strict invariance model did not significantly differ from the scalar model [χ^2^_*diff*_(14) = 16.11, *p* = 0.30]. Finally, we constrained the variances and covariances of the three latent factors across groups (factor invariance) and this model significantly differed from the strict model [χ^2^_*diff*_ (6) = 25.98, *p* < 0.001]. The Lagrange multiplier test approached significance [χ^2^(49) = 65.83, *p* = 0.06], but the univariate chi-square test for the variance and covariance of factor 3 was significant (chi-square of 15.05, *df* of 1, and *p* < 0.001). After freeing Factor 3’s variance and covariance, the factor invariance model did not significantly differ from the strict model [χ^2^_*diff*_(5) = 10.28, *p* = 0.07]. Table 6 presents the completely standardized freed parameters for both groups. Note that the parameter estimate for Factor 3 represents the latent factors’ unstandardized variances.

**TABLE 6:** Parameters without sufficient invariance.

	Intercept 11	Intercept 13	Intercept 15	Intercept 16	Intercept 17	Intercept 24	Residual 2	Residual 6	Residual 7	Residual 13	Residual 15	Factor 3
Controls (*N* = 295)	1.30	0.50	0.91	0.67	0.91	0.89	0.69	0.62	0.87	0.78	0.59	0.23
SDIs (*N* = 320)	1.53	0.59	1.00	0.46	0.69	1.37	0.74	0.69	0.90	0.85	0.79	0.13

**SDIs, substance dependent individuals.**

### Gender and Group Differences

After establishing the MI of the scale, we examined gender differences in LSRP total score and the factor scores in Brinkley’s 3-factor solution. Male SDIs had higher full-scale LSRP scores than female SDIs but the effect size was of medium strength. Male controls and SDIs displayed significantly higher scores than females on Brinkley’s *egocentric* factor. On Brinkley’s *antisocial* factor, female controls had significantly higher mean scores than male controls (*p* < 0.02), whereas for SDIs, males had higher scores than females, but the difference was not significant (*p* < 0.60). Regarding the *callous* factor, males had higher scores than females in both controls and SDIs. Notably, the effect sizes for the *egocentric* and *callous* subscale differences were large. Table 7 presents group means, *SD*s, *t*-values, *p*-values, and effect sizes (Cohen’s d).

**TABLE 7:** Results from *t*-tests for gender differences.

Scale	Gender	Controls				SDIs			
		*M*	*SD*	t/p	Effect size	*M*	*SD*	t/p	Effect size
Brinkley total score	Male	23.62	9.69	3.25/0.001	0.38	30.07	10.35	4.80/0.001	0.62
	Female	20.00	9.25			23.83	9.72		
Brinkley egocentric	Male	9.39	5.65	5.18/0.001	0.59	11.22	5.76	4.58/0.001	0.59
	Female	6.41	4.25			7.94	5.16		
Brinkley antisocial	Male	4.41	2.89	−2.28/0.023	0.27	6.85	3.14	0.52/0.602	0.07
	Female	5.22	3.14			6.65	2.66		
Brinkley callous	Male	2.95	2.87	3.37/0.001	0.38	3.81	2.39	4.70/0.001	0.60
	Female	1.97	2.15			2.43	1.98		

*SDIs, substance dependent individuals. Effect sizes were calculated following Cohen’s (1994) formulas.*

We also compared the factor scores across the control group and the three SDIs subgroups using ANOVA. Tukey’s *post hoc* comparisons showed that control participants had lower scores on Brinkley’s *egocentric* and *callous* factors than HDIs and PDIs. All substance dependent groups scored significantly higher than controls on both the *antisocial* factor and the total LSRP score. There were no significant group differences between heroin, amphetamine, and polysubstance dependent groups on the total LSRP scale and its three factors. Table 8 presents group means, SDs, *F*-values, *p*-values, and group contrasts based on Tukey’s *post hoc* comparisons.

**TABLE 8:** Descriptive statistics and group differences in LSRP factors and total score.

	Control group		HDIs		ADIs		PDIs			
	Mean	*SD*	Mean	*SD*	Mean	*SD*	Mean	*SD*	F/*p*	Contrast
Brinkley total score	15.36	7.83	20.57	8.98	19.40	8.38	21.46	8.12	21.87/0.001	C < HDIs, ADIs, PDIs
Brinkley egocentric	8.08	5.29	10.42	6.03	9.80	5.96	10.70	5.43	9.00/0.001	C < HDIs, PDIs
Brinkley antisocial	4.77	3.02	6.48	3.04	6.57	2.99	7.24	3.01	24.72/0.001	C < HDIs, ADIs, PDIs
Brinkley callous	2.52	2.62	3.67	2.43	3.02	2.21	3.58	2.39	8.47/0.001	C < HDI, PDI

*C, Control group; HDIs, heroin dependent individuals, ADIs, amphetamine dependent individuals; PDIs, polysubstance dependent individuals.*

### Relationships With Conceptually Related Constructs

The modified tri-factor Brinkley model was chosen to examine the construct (convergent and discriminant) and criterion validity of the LSRP due to its better fit to the data. First, we evaluated LSRP’s construct validity by comparing the LSRP and the PCL:SV by conducting zero-order and partial correlations between the LSRP total and factor scores and the PCL:SV total and factor scores (Table 9). Second, zero-order and partial correlations were calculated between all three LSRP factors, the LSRP total score, and the instruments measuring externalizing and internalizing traits and behaviors (Table 10). Primary psychopathy was measured by the factors *egocentric (F1)* and *callous (F3)*, whereas the *antisocial* factor *(F2)* measured secondary psychopathy (Levenson et al., 1995; Brinkley et al., 2008). All three LSRP subscales were positively correlated with the total score and the two factors of the PCL:SV (Factor 1 = callous/unemotional dimension of psychopathy, Factor 2 = impulsive/antisocial dimension of psychopathy). Individual partial correlation analyses for all three LSRP factors were conducted, controlling for the remaining two factors. As expected, results revealed that the *egocentric* and *callous* LSRP factors were positively correlated with PCL:SV callous/unemotional dimension of psychopathy (Factor 1), but not with PCL:SV impulsive/antisocial dimension of psychopathy (Factor 2). In contrast, the LSRP *antisocial* factor was positively related to the PCL:SV impulsive/antisocial dimension of psychopathy (Factor 2), but negatively related to the PCL:SV callous/unemotional dimension of psychopathy (Factor 1). The correlations between the three LSRP factors and the PCL:SV factors were moderate (*r* ∼0.24–0.42) and revealed the expected pattern for both primary and secondary psychopathy. The total score of the LSRP exhibited the highest correlation with the PCL:SV total score (*r* = 0.48).

**TABLE 9:** Construct validity: Zero-order and partial correlations between LSRP subscales and PCL:SV factors in the total sample.

	Egocentric	Callous	Antisocial	Total
PCL:SV factor 1	0.40** (0.26**)	0.35** (0.13**)	0.24** (-0.18*)	0.45**
PCL:SV factor 2	0.32** (-0.08)	0.28** (0.04)	0.42** (0.37**)	0.45**
PCL:SV total	0.38** (0.17**)	0.33** (0.16**)	0.37** (0.30**)	0.48**

PCL:SV F1, Psychopathy Checklist: Screening Version Factor 1; PCL:SV F2, Psychopathy Checklist: Screening Version Factor 2; PCL:SV Total, Psychopathy Checklist: Screening Version Total Score. Zero-order correlations are outside the parentheses, partial correlations are inside the parentheses, values in bold are significant: **at the level of 0.01 (2-tailed), *at the level of 0.05 (2-tailed).

**TABLE 10:** Convergent and discriminant validity: Zero-order and partial correlations between LSRP scales and criterion variables in the total sample.

Criterion variables	Egocentric	Callous	Antisocial	Total
**Antisocial behavior**				
Conduct disorder	0.21** (0.09*)	0.21** (0.13**)	0.26** (0.19**)	0.29**
Antisocial personality disorder	0.29** (0.12**)	0.29** (0.20**)	0.33** (0.25**)	0.40**
**Aggression**				
**AQ total**	0.35** (0.11*)	0.22** (0.10)	0.59** (0.57**)	0.51**
Physical aggression	0.32** (0.13*)	0.26** (0.14**)	0.43** (0.41**)	0.45**
Verbal aggression	0.18** (0.03)	0.12** (0.04)	0.32** (0.26**)	0.27**
Anger	0.21** (−0.04)	0.12** (0.00)	0.58** (0.58**)	0.39**
Hostility	0.34** (0.20*)	0.16** (0.08)	0.38** (0.31**)	0.41**
**Internalizing psychopathology**				
Anxiety sensitivity	0.20** (0.13*)	0.04 (−0.01)	0.33** (0.29**)	0.26**
State anxiety	0.16** (0.04)	0.12** (0.02)	0.34** (0.30**)	0.27**
Trait anxiety	0.15** (−0.03)	0.10** (0.00)	0.49** (0.43**)	0.31**
Depression	0.21** (0.06)	0.15** (0.06)	0.41** (0.33**)	0.34**
Alexithymia	0.32** (0.21**)	0.19** (0.07)	0.36** (0.28**)	0.41**
**Impulsivity**				
*BIS-11 total*	0.24** (0.02)	0.14** (0.00)	0.49** (0.48**)	0.38**
Non-planning impulsivity	0.17** (−0.02)	0.14** (−0.01)	0.40** (0.38**)	0.30**
Motor impulsivity	0.23** (0.05)	0.09* (0.01)	0.38** (0.39**)	0.32**
Attentional impulsivity	0.21** (0.01)	0.11** (0.00)	0.46** (0.43**)	0.34**
*SSS-V total*	0.24** (0.15**)	0.11** (−0.02)	0.20** (0.15**)	0.27**
Disinhibition	0.36** (0.28**)	0.17** (0.01)	0.21** (0.14**)	0.37**
Boredom susceptibility	0.22** (0.14**)	0.13** (0.03)	0.20** (0.13**)	0.26**
Thrill and adventure seeking	0.11** (0.07)	0.04 (−0.02)	0.09* (0.07)	0.12**
Experience seeking	0.00 (−0.06)	−0.02 (−0.06)	0.09* (0.10*)	0.03
*UPPS total*	0.16** (0.06)	0.15** (0.12*)	0.46** (0.51**)	0.33**
Negative urgency	0.22** (−0.04)	0.14** (0.03)	0.58** (0.57**)	0.40**
Positive urgency	0.33** (0.15**)	0.23** (0.14**)	0.49** (0.43**)	0.47**
Premeditation (lack of)	0.04 (−0.12*)	0.15** (0.15**)	0.25** (0.22**)	0.17**
Perseverance (lack of)	0.09* (−0.09)	0.12** (0.07)	0.37** (0.35**)	0.23**
Sensation seeking	0.28** (0.18**)	0.12** (0.01)	0.21** (0.14**)	0.30**
*WURS/ADHD total*	0.12** (−0.10)	0.06 (−0.01)	0.46** (0.44**)	0.26**
**Demographics**				
Age	−0.15** (−0.15**)	−0.07 (−0.06)	−0.04 (−0.01)	−0.13**
IQ (Raven matrices)	−0.18** (−0.01)	−0.24** (−0.26**)	−0.13** (−0.09)	−0.24**
Years of education	−0.19** (−0.17**)	−0.17** (−0.11*)	−0.13** (−0.08)	−0.22**
Gender	−0.29** (−0.23**)	−25** (−0.16**)	−0.01 (0.07)	−0.27**

AQ Total, Aggression Questionnaire Total Score; BIS-11 Total, Barratt Impulsiveness Scale–11 Total Score; SSV-V Total, Sensation Seeking Scale-V Total Score; UPPS Total, UPPS-P Impulsive Behavior Scale Total Score; WURS Total, Wender Utah Rating Scale Total Score. Zero-order correlations are outside the parentheses, partial correlations are inside the parentheses, values in bold are significant: **at the level of 0.01 (2-tailed), *at the level of 0.05 (2-tailed).

With regards to the convergent validity of the LSRP and its subscales, partial correlations revealed as expected that the *antisocial* factor of the LSRP was positively related to measures of antisocial behavior (CD, ASPD), all forms of aggression measured by the AQ, all measures of internalizing psychopathology (anxiety, anxiety sensitivity, depression, and alexithymia), and most measures of impulsivity, except thrill and adventure seeking from the SSS. With regards to discriminant validity, as expected, measures of internalizing psychopathology did not correlate with any of the two primary LSRP psychopathy factors (*egocentric* and *callous*), except the correlations of anxiety sensitivity and alexithymia with the *egocentric* factor of the LSRP. The *egocentric* factor was positively related to measures of antisocial behavior (CD, ASPD), physical aggression and hostility (AQ), and sensation seeking (SSS, UPPS). The *callous* factor of the LSRP was positively correlated to CD, ASPD, and physical aggression (AQ). Both the *egocentric* and the *callous* factors were positively associated with positive urgency (UPPS), indicating that high levels of egocentricity and callousness are related to impulsive behaviors in response to positive affective states. In addition, the two primary psychopathy factors demonstrated differential patterns of relationships with the lack of premeditation subscale of the UPPS, with the *egocentric* factor related to higher ability to delay action in favor of planning, whereas the *callous* factor was positively related to lack of premeditation.

With regards to criterion validity, relevant demographic characteristics including age, estimated IQ, years of education, and gender were negatively associated with the *egocentric* and *callous* factors, but not with the *antisocial* factor at the partial correlation level. As expected, the two LSRP primary psychopathy factors were negatively related to female gender in our study.

There were group differences in the patterns of correlations between the LSRP and the validation variables between SDIs and non-dependent controls (Tables 11, 12). The *egocentric* factor was positively associated to hostility (AQ), alexithymia, and disinhibition (SSS) and negatively associated to age in both control and substance-dependent groups. However, in SDIs the *egocentric* subscale of the LSRP was positively correlated with depression, boredom susceptibility (SSS), positive urgency (UPPS), and negatively related to symptoms of ADHD, whereas in the control group the *egocentric* factor was positively related to anxiety sensitivity and negatively related to female gender and years of education. The *antisocial* subscale was related to all dimensions of aggression (AQ), all measures of internalizing psychopathology (STAY, TAS-20, ASI, BDI-II), all measures of impulsivity (BIS, UPPS), and symptoms of ADHD in both groups. In the control group, the *antisocial* factor was further positively related to female gender and negatively related to IQ. In SDIs, the *antisocial* factor was uniquely related to antisocial behavior (CD and ASPD) and boredom susceptibility (SSS). The *callous* factor was positively related to antisocial behavior (ASPD) and negatively related to female gender in both control and substance-dependent groups. In the control group, the *callous* factor was further positively correlated with hostility (AQ) and lack of premeditation (UPPS) and negatively related to IQ and years of education, whereas in SDIs the *callous* subscale was uniquely related to physical aggression (AQ) and positive urgency (UPPS). For convergent and discriminant validity of the LSRP subscales across groups of SDIs, please see the Supplementary Material of the study (Supplementary Tables S1–S4).

**TABLE 11:** Convergent and discriminant validity: Zero-order and partial correlations between LSRP scales and criterion variables in the control group.

Criterion variables	Egocentric	Callous	Antisocial	Total
**Antisocial behavior**				
Conduct disorder	0.17** (0.12*)	0.08 (0.04)	0.13** (0.08)	0.19**
Antisocial personality disorder	0.22** (0.14*)	0.22** (0.18**)	0.15** (0.10)	0.28**
**Aggression**				
**AQ total**	0.30** (0.03)	0.13* (0.08)	0.60** (0.60**)	0.48**
Physical aggression	0.29** (0.09)	0.14* (0.01)	0.29** (0.29**)	0.36**
Verbal aggression	0.06 (−0.06)	0.09 (0.07)	0.28** (0.25**)	0.18**
Anger	0.15* (−0.10)	0.02 (−0.02)	0.54** (0.57**)	0.31**
Hostility	0.36** (0.20**)	0.13* (0.16*)	0.49** (0.46**)	0.48**
**Internalizing psychopathology**				
Anxiety sensitivity	0.13* (0.14*)	0.04 (0.03)	0.27** (0.28**)	0.21**
State anxiety	0.10 (0.02)	0.09 (0.04)	0.33** (0.31**)	0.23**
Trait anxiety	0.07 (−0.09)	0.02 (−0.02)	0.52** (0.49**)	0.25**
Depression	0.09 (−0.04)	0.07 (0.07)	0.40** (0.37**)	0.24**
Alexithymia	0.29** (0.17*)	0.12 (0.08)	0.36** (0.31**)	0.38**
**Impulsivity**				
*BIS−11 total*	0.12* (−0.08)	0.04 (−0.03)	0.45** (0.45**)	0.27**
Non−planning impulsivity	0.07 (−0.07)	0.06 (−0.02)	0.34** (0.34**)	0.19**
Motor impulsivity	0.13* (−0.02)	−0.01 (−0.04)	0.33** (0.33**)	0.21**
Attentional impulsivity	0.11 (−0.11)	0.05 (0.01)	0.45** (0.45**)	0.26**
*SSS−V total*	0.17** (0.08)	0.00 (−0.11)	0.09 (0.05)	0.15**
Disinhibition	0.30** (0.17*)	0.08 (−0.04)	0.13* (0.08)	0.28**
Boredom susceptibility	0.11 (0.03)	0.06 (−0.01)	0.06 (−0.02)	0.15*
Thrill and adventure seeking	0.12* (0.09)	−0.03 (−0.12)	0.07 (0.05)	0.09
Experience seeking	−0.05 (−0.09)	−0.09 (−0.11)	0.01 (0.02)	−0.06
*UPPS total*	0.12* (0.02)	0.09 (0.04)	0.48** (0.49**)	0.29**
Negative urgency	0.15* (−**0.08)**	−0.01 (−0.08)	0.58** (0.57**)	0.32**
Positive urgency	0.23* (0.10)	0.08 (0.06)	0.48** (0.45**)	0.38**
Premeditation (lack of)	0.01 (−0.11)	0.16* (0.16*)	0.18** (0.14*)	0.12*
Perseverance (lack of)	−0.02 (−0.13)	0.08 (0.07)	0.33** (0.31**)	0.13*
Sensation Seeking	0.31* (0.20**)	0.06 (−0.06)	0.17** (0.12)	0.29**
*WURS/ADHD total*	0.08 (−0.06)	−0.04 (−0.05)	0.42** (0.34**)	0.21**
**Demographics**				
Age	−0.18* (−0.14*)	−0.09 (−0.05)	−0.02 (0.04)	−0.16**
IQ (Raven matrices)	−0.13* (−**0.02)**	−0.28** (−0.36**)	−0.11 (−0.16*)	−0.22**
Years of education	−0.28** (−0.29**)	−0.17** (−0.14*)	−0.05 (−0.03)	−0.26**
Gender	−0.28** (−0.29**)	−0.19** (−0.14*)	0.13* (0.19*)	−0.20**

AQ Total, Aggression Questionnaire Total Score; BIS−11 Total, Barratt Impulsiveness Scale–11 Total Score; SSV-V Total, Sensation Seeking Scale-V Total Score; UPPS Total, UPPS-P Impulsive Behavior Scale Total Score; WURS Total, Wender Utah Rating Scale Total Score. Zero-order correlations are outside the parentheses, partial correlations are inside the parentheses, values in bold are significant: **at the level of 0.01 (2-tailed), *at the level of 0.05 (2-tailed).

**TABLE 12:** Convergent and discriminant validity: Zero-order and partial correlations between LSRP scales and criterion variables in the substance-dependent (SDI) group.

Criterion variables	Egocentric	Callous	Antisocial	Total
**Antisocial behavior**				
Conduct disorder	0.15** (0.04)	0.20** (0.14*)	0.17** (0.12*)	0.21**
Antisocial personality disorder	0.21** (0.09)	0.26** (0.18**)	0.20** (0.13**)	0.29**
**Aggression**				
**AQ total**	0.31** (0.15)	0.22** (0.09)	0.51** (0.48**)	0.45**
Physical aggression	0.26** (0.09)	0.27** (0.20**)	0.38** (0.39**)	0.39**
Verbal aggression	0.22** (0.12)	0.09 (−0.03)	0.28** (0.23**)	0.27**
Anger	0.17** (0.01)	0.13* (0.01)	0.54** (0.53**)	0.35**
Hostility	0.30** (0.24**)	0.17** (0.04)	0.28** (0.17*)	0.35**
**Internalizing psychopathology**				
Anxiety sensitivity	0.21* (0.13)	−0.02 (−0.08)	0.33** (0.25**)	0.25**
State anxiety	0.17** (0.07)	0.11 (0.01)	0.30** (0.29**)	0.26**
Trait anxiety	0.18** (0.05)	0.16** (0.08)	0.44** (0.34**)	0.33**
Depression	0.28** (0.22**)	0.18** (0.07)	0.38** (0.29**)	0.38**
Alexithymia	0.33** (0.25**)	0.25** (0.07)	0.34** (0.26**)	0.41**
**Impulsivity**				
*BIS−11 total*	0.24** (0.09)	0.13* (−0.01)	0.42** (0.43**)	0.35**
Non−planning impulsivity	0.16** (0.01)	0.12* (−0.03)	0.33** (0.33**)	0.26**
Motor impulsivity	0.21** (0.08)	0.09 (0.03)	0.30** (0.36**)	0.28**
Attentional impulsivity	0.23** (0.14)	0.10 (−0.02)	0.42** (0.37**)	0.34**
*SSS−V total*	0.23** (0.19*)	0.13* (0.05)	0.15** (0.13)	0.25**
Disinhibition	0.34** (0.36**)	0.19** (0.01)	0.16** (0.11)	0.35**
Boredom susceptibility	0.25** (0.23**)	0.14* (0.02)	0.23** (0.24**)	0.29**
Thrill and adventure seeking	0.05 (−0.01)	0.05 (0.14)	0.01 (−0.03)	0.05
Experience seeking	−0.03 (−0.05)	−0.04 (−0.05)	0.04 (0.08)	−0.02
*UPPS total*	0.11* (0.04)	0.13* (0.17*)	0.35** (0.41**)	0.24**
Negative urgency	0.18** (−0.05)	0.19** (0.12)	0.48** (0.47**)	0.34**
Positive urgency	0.32** (0.15*)	0.31** (0.19*)	0.36** (0.30**)	0.43**
Premeditation (lack of)	0.01 (−0.15)	0.08 (0.10)	0.24* (0.25**)	0.12*
Perseverance (lack of)	0.11* (−0.06)	0.11 (0.04)	0.34** (0.36**)	0.23**
Sensation Seeking	0.19** (0.14)	0.09 (0.07)	0.10 (0.03)	0.19**
*WURS/ADHD total*	0.01 (−0.18*)	0.01 (−0.07)	0.37** (0.40**)	0.14*
**Demographics**				
Age	−0.14* (−0.18*)	−0.04 (−0.07)	−0.09 (−0.13)	−0.13*
IQ (Raven matrices)	−0.21** (−0.04)	−0.18** (−0.13)	−0.12* (−0.03)	−0.24**
Years of education	−0.02 (−0.03)	−0.08 (−0.06)	−0.06 (−0.03)	−0.06
Gender	−0.25** (−0.14)	−0.26** (−0.15*)	−0.03 (0.04)	−0.25**

AQ Total, Aggression Questionnaire Total Score; BIS-11 Total, Barratt Impulsiveness Scale–11 Total Score; SSV-V Total, Sensation Seeking Scale-V Total Score; UPPS Total, UPPS-P Impulsive Behavior Scale Total Score; WURS Total, Wender Utah Rating Scale Total Score. Zero-order correlations are outside the parentheses, partial correlations are inside the parentheses, values in bold are significant: **at the level of 0.01 (2-tailed), *at the level of 0.05 (2-tailed).

## Discussion

The aims of the present study were to examine the factor structure and psychometric properties of the Bulgarian version of the LSRP in a community sample, explore its external validity, and evaluate its ability to distinguish between SDIs and non-dependent controls. In line with our predictions, the three-factor model of the LSRP (Brinkley et al., 2008) showed the best fit in the current sample. The internal consistency of the scale and its subscales were acceptable. With regards to its diagnostic utility, the LSRP was moderately correlated with the PCL:SV total score. The LSRP *egocentric* and *callous* factors were correlated with the PCL:SV callous/unemotional dimension of psychopathy, whereas the LSRP *antisocial* factor was related to the PCL:SV impulsive/antisocial dimension of psychopathy. The instrument correlated well with theoretically related variables, suggesting acceptable convergent and discriminant validity of the Bulgarian version of the LSRP. All groups of SDIs had higher scores than control participants on the LSRP total score and subscales, indicating that the LSRP can discriminate well between the control group and the group of SDIs.

Research reveals inconsistencies across samples and studies with regards to the latent factor structure of the LSRP, with some studies supporting the original two-factor structure (Levenson et al., 1995; Lynam et al., 1999), whereas others report a better fit for a three-factor model of psychopathy (Brinkley et al., 2008; Sellbom, 2011; Somma et al., 2014; Shou et al., 2017; Garofalo et al., 2018). In line with previous research (e.g., Lynam et al., 1999; Brinkley et al., 2008; Sellbom, 2011; Shou et al., 2017; Garofalo et al., 2018), we did not replicate the original two-factor structure of the LSRP, even when we included the inter-correlating errors, as suggested by Lynam et al. (1999). This was not surprising because the use of multiple modifications in confirmatory factor analysis based on empirical and not theoretical grounds may lead to overfitting and difficulty replicating in other samples (MacCallum, 1986; Silvia and MacCallum, 1988). We successfully replicated Brinkley et al.’s (2008) three-factor model with its *egocentric*, *antisocial*, and *callous* factors, with only four inter-correlating errors, which were accepted based on theoretical grounds. The model included 19 out of the original 26 items. Although Brinkley et al.’s (2008) study was based entirely on females, recent studies using mixed samples have successfully replicated the three-factor structure (Sellbom, 2011; Somma et al., 2014; Shou et al., 2017; Garofalo et al., 2018). Similarly, our data indicate that the three-factor model of the LSRP fits well in a community sample of male and female SDIs and non-substance-dependent control participants.

In terms of internal consistency, the total scores of the LSRP and the *egocentric* subscale (F1) had high reliability in both SDIs and controls (α = 0.78–0.81). The internal consistencies of the *callous* (F3) and *antisocial* (F2) subscales were lower, ranging from poor to acceptable across groups (α = 0.52–0.69), and thus were below the accepted cutoff value of 0.70. These results were similar to previous studies, which have consistently found a higher alpha coefficient for the *egocentric* subscale and lower alpha coefficients for the *callous* and *antisocial* subscales of the LSRP (Brinkley et al., 2008; Shou et al., 2017). However, Cronbach’s alpha is highly affected by the number of test items and is consistently lower in shorter scales (the *callous* subscale has only four items and the *antisocial* subscale consisted of five items, whereas the *egocentric* subscale included 10 items). Therefore, it has been argued that Cronbach’s alphas between 0.60 and 0.70 may be regarded as adequate values of internal consistency for shorter scales (Clark and Watson, 1995). According to these standards, the LSRP subscales could be regarded as reliable in our sample, with the exception of the *callous* and *antisocial* subscales in the group of SDIs (Cronbach’s alphas were 0.52 and 0.57, respectively). Therefore, we also examined the mean inter-item correlations, suggested to be a more sensitive measure of internal consistency and scale homogeneity (Clark and Watson, 1995). In our sample, the mean inter-item correlations of all three LSRP subscales fell between 0.21 and 0.36 across groups, which indicated acceptable internal consistency as proposed by Clark and Watson (1995). In general, our results suggest that the LSRP and its subscales were all internally consistent in our sample of SDIs and non-dependent community volunteers.

We also conducted measurement invariance (MI) analyses to verify that the LSRP reliably and similarly measures self-reported psychopathy among SDIs and non-dependent controls. The MI analyses showed that the LSRP is slightly invariant across SDIs and control participants with regards to six intercepts and five residuals. Item 17 (“I find myself in the same kind of trouble, time after time”) had an intercept favoring the controls, whereas item 24 (“I have been in a lot of shouting matches with other people”) had an intercept favoring SDIs. Since both items 17 and 24 belong to the *antisocial* factor and their intercepts are balanced and did not favor either group, the invariance for the latent *antisocial* factor can be considered unsubstantial. In contrast, item 11 (“I often admire a really clever scam”) and item 13 (“I enjoy manipulating other people’s feelings”), which both fall into the *egocentric* subscale, had intercepts favoring SDIs. These results suggest that the *egocentric* factor is slightly invariant across groups with SDIs scoring higher than controls. Item 15 (“Even if I were trying very hard to sell something, I wouldn’t lie about it”) had both differing intercept and residual, and the intercept favored the controls. In contrast, item 16 (“Cheating is not justified because it is unfair to others”) had an intercept favoring SDIs. Taking into consideration that both items 15 and 16 fall into the *callous* factor and that their intercepts did not favor neither the controls nor the SDIs, the invariance for the latent *callous* factor should not be substantial. This is confirmed by the latent mean for SDIs being slightly below (0.24 points) the latent mean for controls. Thus, except for SDIs scoring slightly more egocentric and less callous on several items as compared to controls, the LSRP seems to function invariantly across the two samples and putatively the populations that they represent. A possible explanation for SDIs scoring less callous than controls might be that the majority of SDIs in our sample underwent therapy and were in protracted abstinence at the time of testing. It is possible that the therapeutic work with SDIs has led to reduction in manipulative and callous behaviors, which are common in the context of addiction. Alternatively, therapy could also increase the awareness to socially desirable answering style, which may explain, in part, the lower scores on some items reported by SDIs. However, comparisons between the control group and the substance-dependent groups revealed that all SDIs (HDIs, ADIs, and PDIs) scored significantly higher on the LSRP total score, indicating elevated levels of psychopathy. As our study sample consisted primarily of SDIs in protracted abstinence, these findings suggest that psychopathy does not alleviate with abstinence and may be a stable personality dimension of SDIs, regardless of their drug preference. In light of the chronic relapsing nature of SUDs, studies suggest that SDIs are at risk for relapse even after substantial periods of full remission (Xie et al., 2005). Given that comorbid psychopathy is recognized as a robust risk factor for relapse in SDIs (Smith and Newman, 1990; Alterman et al., 1998; O’Neill et al., 2003; Richards et al., 2003), the elevated levels of psychopathy found in SDIs in protracted abstinence may place them at increased risk for future relapse, even after sustained remission. These findings emphasize the growing need of developing personality-tailored interventions that may decrease the long-term risk of relapse in specific vulnerable groups of SDIs.

With regards to its diagnostic utility, previous assessments of the psychometric properties of the LSRP suggest promising criterion-related validity, indicated by its moderate relationships with other measures of psychopathy (Lynam et al., 1999; Brinkley et al., 2001). In the current study, all three LSRP factors demonstrated the expected patterns of correlations with the PCL:SV factors. The LSRP *egocentric* and *callous* subscales were positively related only to the PCL:SV Factor 1, measuring the callous/unemotional dimension of psychopathy. The LSRP *antisocial* subscale was positively related to the impulsive/antisocial dimension of the PCL:SV (Factor 2) and negatively related to the PCL:SV Factor 1. In our sample, the correlation between the total scores on the PCL:SV and the LSRP (*r* = 0.48) was higher than those reported previously (*r* = 0.30–0.35; Brinkley et al., 2001; Poythress et al., 2010). This difference could be due to the use of the PCL:SV in the current study in contrast to the PCL-R in previous studies. An item response theory analysis of the two PCL versions by Cooke et al. (1999) revealed that the items in the PCL:SV have equal or even greater discriminating value compared to the corresponding items in PCL-R, which in turn could explain the higher correlations with the LSRP total scores in the current study.

Overall, the LSRP’s subscales exhibited the expected patterns of inter-correlations with other measures of externalizing and internalizing psychopathology, as well as with measures of impulsivity. As expected, the *antisocial* subscale of the LSRP (reflecting secondary psychopathy) was related to elevated levels of aggression, impulsivity, internalizing psychopathology (anxiety, anxiety sensitivity, depression, alexithymia) and externalizing psychopathology (CD, ASPD, ADHD). Our results suggest that the *antisocial* factor of the LSRP maps quite well onto the original conceptualization of secondary psychopathy reflecting impulsive and antisocial lifestyle and higher levels of emotional lability (Karpman, 1948; Levenson et al., 1995).

The convergent and discriminant validity of the *egocentric* and *callous* subscales (reflecting primary psychopathy) were more questionable, as some unexpected relationships with external variables emerged. Our data did, however, indicate that the *egocentric* and *callous* subscales were related to some theoretically relevant external variables. For example, in line with expectations, higher scores on the *egocentric* and *callous* factors were associated with antisocial behaviors (CD, ASPD) and higher levels of self-reported physical aggression, supporting the theoretical and empirical conceptualizations of primary psychopathy being associated with specific emotional deficits related to cruel disregard of the rights of others (Frick et al., 2005). Our results are also consistent with previous research on the psychometric properties of the LSRP indicating that the *egocentric* and *callous* factors are related to elevated levels of antisocial and aggressive behavior (Brinkley et al., 2008; Somma et al., 2014; Garofalo et al., 2018).

In addition, the *callous* and *egocentric* factors were surprisingly related to measures of impulsivity (lack of premeditation and positive urgency). In the literature, primary psychopathy has consistently been related to more calculated and premeditated actions as compared to the much more disinhibited and impulsive secondary psychopathy (for review, see Poythress and Hall, 2011). In our study, only the *egocentric* factor was related to higher ability to delay action in favor of planning, whereas the *callous* factor was related to tendency toward more impulsive actions. In addition, the *egocentric* factor was related to measures of sensation seeking (disinhibition and boredom susceptibility), in line with previous studies on the validity of the LSRP (Levenson et al., 1995; Brinkley et al., 2008; Sellbom, 2011). The *egocentric* and *callous* factors were also associated with positive urgency (measure of emotional impulsivity). Although unexpected, these findings suggest that individuals with high levels of primary psychopathy (reflecting manipulative, egocentric, and callous traits) could be more impulsive in response to positive affective states or when anticipating reward. This notion is consistent with previous conceptualizations of the primary psychopath as showing little concern about the possible negative consequences of behavior when anticipating personal gain (Skeem et al., 2007).

Regarding measures of internalizing psychopathology, we hypothesized that the *egocentric* and *callous* subscales (primary psychopathy) would be negatively related or unrelated to measures of internalizing traits and behaviors. Our results show that in line with expectations the *callous* subscale was not related to any measures of internalizing psychopathology, indicating good discriminant validity. Surprisingly, the *egocentric* factor was positively related to anxiety sensitivity and alexithymia in the total sample. These correlations were unexpected because the LSRP *egocentric* subscale is composed of items reflecting manipulative tendencies, lack of remorse, and striving for personal gain, which are among the main characteristics of primary psychopathy that has been consistently unrelated or negatively related to measures of internalizing psychopathology (Skeem et al., 2003; Hicks et al., 2004; Poythress and Skeem, 2006). Although unexpected, the correlations of the *egocentric* subscale with measures of internalizing psychopathology were similar to those reported by other studies of the LSRP in community samples (Shou et al., 2017). One possible explanation is that the associations between egocentric traits and measures of internalizing psychopathology in our sample consisting of SDIs and non-dependent community volunteers could reflect tendencies more typical of community life. Nevertheless, our findings were counterintuitive and may question the construct validity of the *egocentric* subscale of the LSRP in Bulgarian community samples.

Overall, the patterns of relationships between the LSRP factors and external variables in our study were influenced by the specific characteristics of our sample, which consisted of individuals with past history of SUDs and individuals from the general population. The LSRP subscales displayed some common but also some unique patterns of inter-correlations with external variables in SDIs and non-dependent controls. For example, the *callous* factor was related to antisocial behavior in both groups but was uniquely associated with physical aggression in SDIs and with hostility in non-dependent controls. In addition, callous traits were related to poor ability to delay action in favor of planning (lack of premeditation) in the control group, whereas in the substance-dependent group callous traits were related to impulsive behaviors in response to positive affective states (positive urgency). The *antisocial* factor was associated with aggression, impulsivity, sensation seeking, and internalizing traits (anxiety, anxiety sensitivity, depression, and alexithymia) in both groups, but it was uniquely related to antisocial behavior (CD/ASPD) in SDIs. In contrast, the *egocentric* factor was distinctively associated with antisocial behavior (CD/ASPD) in community controls. These results suggest that antisocial behavior is motivated by distinct personality traits in non-dependent individuals from the community and in SDIs. Our findings suggest that personality features such as manipulativeness and antagonism (egocentric factor) are more strongly predictive of antisocial behavior in the community than in SDIs.

In summary, the LSRP subscales exhibited the expected correlations with theoretically related external variables, suggesting that the Bulgarian version of the LSRP has acceptable convergent and discriminant validity. In addition, the differential patterns of associations of the three LSRP factors with external criteria suggest that the three subscales of the LSRP were successful at measuring distinguishable aspects of psychopathy. Thus, our findings provide further support to the multi-factor model of psychopathy and encourage future research to further examine the patterns of correlations, specific to different aspects of psychopathy, and in different samples of individuals.

### Limitations and Future Research

There are some limitations of our study that are worth noting. First, our results could be influenced by cultural differences. Psychopathy has typically been studied in North America and Western Europe, and studies on its generalizability and manifestations among individuals of different cultures are scarce. To our knowledge, there are no studies on the psychometric properties of the LSRP in Eastern Europe. However, the PCL-R has proven to be a reliable tool for measuring psychopathy in Turkey (Tutuncu et al., 2015), and the PCL:SV has been successfully validated in Lithuania (Žukauskienė et al., 2010) and Bulgaria (Wilson et al., 2014).

Further, most of the instruments we used were self-report measures; therefore, our results may be vulnerable to subjective and cultural factors. Future research could use clinical interviews and neurocognitive tasks for the assessment of conceptually relevant external variables in order to provide more valid and objective measures of external validity. In addition to the possible increase in validity, examining the neurocognitive correlates of the LSRP factors may increase their predictive heuristic value.

## Notes

Note that intercepts and residuals are in the observed metric of LSRP (i.e., Strongly Disagree (1) to Strongly Agree (4) Likert scale), whereas the factor mean differences are standardized.

## Data Availability Statement

The datasets generated for this study are available on request to the corresponding author.

## Ethics Statement

The studies involving human participants were reviewed and approved by Virginia Commonwealth University (VCU) Institutional Review Board and Medical University – Sofia, Institutional Review Board. The participants provided their written informed consent to participate in this study.

## Author Contributions

EP, GY, KB, and JV conceived the study. GY performed the statistical analyses and drafted the analysis and results sections. EP drafted the introduction and discussion sections. EP, KB, and GV collected and managed the data with the assistance of the Bulgarian Addiction Institute’s research team. VP consulted on the statistical analyses. JV supervised the data collection and analyses and drafted portions of the manuscript. All authors discussed the results and contributed to the final manuscript.

## Conflict of Interest

GV discloses that he has ownership interests in the Bulgarian Addictions Institute, where data collection took place. The remaining authors declare that the research was conducted in the absence of any commercial or financial relationships that could be construed as a potential conflict of interest.

## Footnotes

1 Factor analyses and measurement invariance analyses were conducted with R’s package *lavaan* (Rosseel, 2012).
