# Supplementary material for: Validation of the Levenson Self-Report Psychopathy Scale in Bulgarian Substance-Dependent Individuals
Source: Front Psychol. 2020 Jun 9;11:1110. doi: 10.3389/fpsyg.2020.01110 (PMC7296064; doi:10.3389/fpsyg.2020.01110)
Supplement: Supplementary file 1 [file Data_Sheet_1.docx]

**Table S1.** Convergent and discriminant validity: Zero-order and partial correlations between LSRP scales and criterion variables in heroin-dependent individuals.

| **Criterion Variables** | **Egocentric** | **Callous** | **Antisocial** | **Total** |  |
| --- | --- | --- | --- | --- | --- |
| **Antisocial behavior** | | | | |  |
| Conduct disorder | .02 (-.07) | .12 (.12) | .12 (.11) | .08 |  |
| Antisocial personality disorder | **.20*** (.06) | .13 (.06) | **.30** (.24*)** | **.27**** |  |
| **Aggression** | | | | | |
| **AQ total** | **.28**** (.14) | **.21**** (.29) | **.47**** (.29) | **.41**** |  |
| Physical aggression | .19 (.13) | **.27** (.35*)** | **.37**** (.29) | **.32**** |  |
| Verbal aggression | .17 (.29) | .02 (.06) | .16 (-.01) | .18 |  |
| Anger | **.26**** (.02) | .17 (.28) | **.53**** (.26) | **.40**** |  |
| Hostility | **.22* (.32*)** | .13 (.12) | **.27**** (.07) | **.28**** |  |
| **Internalizing psychopathology** | | | | |  |
| Anxiety sensitivity | **.26*** (.14) | -.08 (.05) | **.28**** (.06) | **.24*** |  |
| State anxiety | **.22*** (.17) | .11 (.23) | **.29**** (-.14) | **.28**** |  |
| Trait anxiety | **.23*** (.11) | .04 (.07) | **.47**** (.04) | **.33**** |  |
| Depression | **.29** (.39*)** | .17 (.08) | **.41**** (.21) | **.38**** |  |
| Alexithymia | **.41** (.54**)** | **.29*** (.09) | **.31*** (.05) | **.47**** |  |
| **Impulsivity** | | | | |  |
| *BIS-11 total* | **.26**** (.14) | .18 (.12) | **.36**** (.26) | **.34**** |  |
| Nonplanning impulsivity | **.26**** (.19) | **.22*** (.13) | **.33**** (.16) | **.34**** |  |
| Motor impulsivity | .12 (-.01) | .13 (.18) | .17 (.25) | .17 |  |
| Attentional impulsivity | **.26**** (.19) | .07 (-.06) | **.42**** (.23) | **.34**** |  |
| *SSS-V total* | **.29** (.32*)** | **.27**** (.25) | .14 (-.09) | **.31**** |  |
| Disinhibition | **.43** (.52**)** | **.29**** (.05) | .17 (-.03) | **.42**** |  |
| Boredom susceptibility | .15 (.17) | .13 (.01) | **.23**** (.12) | **.21*** |  |
| Thrill and adventure seeking | .10 (.07) | .15 **(.40**)** | -.05 (-.27) | .09 |  |
| Experience seeking | .07 (.04) | .14 (.15) | .04 (-.03) | .09 |  |
| *UPPS total* | .21 (-.02) | .07 **(.32*)** | .19 (.16) | .10 |  |
| Negative urgency | **.21**** (-.07) | .16 (.16) | **.34**** (.12) | **.29**** |  |
| Positive urgency | .24 (.09) | **.36* (.29*)** | .28 (.16) | **.35*** |  |
| Premeditation (lack of) | -13 (-.28) | .12 (.26) | **.21*** (.14) | .02 |  |
| Perseverance (lack of) | .02 (-.22) | .19 (.19) | **.35** (.38**)** | .18 |  |
| Sensation Seeking | **.26**** (.28) | .15 (.17) | .05 (-.21) | **.24*** |  |
| *WURS / ADHD total* | -.09 (-.24) | -.05 (-.05) | **.30** (.33*)** | .02 |  |
| **Demographics** | | | | |  |
| Age | **-.23*** (-.11) | -.12 (-.12) | **-.26**** (-.28) | **-.28**** |  |
| IQ (Raven matrices) | -.19 (-.01) | **-.21*** (-.11) | **-.28** (-.37**)** | **-.28**** |  |
| Years education | .11 (.02) | -.05 (.03) | -.12 (-.17) | .02 |  |
| Gender | **-.28**** (-.19) | **-.38** (-.29*)** | -.06 (.15) | **-.31**** |  |

*Note*: Zero-order correlations are outside the parentheses, partial correlations are inside the parentheses, values in bold are significant: ** at the level of 0.01 (2-tailed), * at the level of 0.05 (2-tailed).

**Table S2.** Convergent and discriminant validity: Zero-order and partial correlations between LSRP scales and criterion variables in amphetamine-dependent individuals.

| **Criterion Variables** | **Egocentric** | **Callous** | **Antisocial** | **Total** |
| --- | --- | --- | --- | --- |
| **Antisocial behavior** | | | | |
| Conduct disorder | **.37**** (.20) | **.37* (.23*)** | **.21**** (.17) | **.44**** |
| Antisocial personality disorder | **.34** (.22*)** | **.32**** (.18) | .02 (-.04) | **.34**** |
| **Aggression** | | | | |
| **AQ total** | **.29**** (.14) | **.23*** (.09) | **.49** (.56**)** | **.45**** |
| Physical aggression | **.30**** (.22) | **.25*** (.10) | **.35** (.41**)** | **.41*** |
| Verbal aggression | .18 (-.03) | .05 (.02) | **.27* (.39**)** | **.24*** |
| Anger | .04 (-.01) | .05 (-.08) | **.55** (.56**)** | **.24*** |
| Hostility | **.37**** (.17) | **.34**** (.22) | **.25*** (.19) | **.44**** |
| **Internalizing psychopathology** | | | | |
| Anxiety sensitivity | .11 (.06) | .00 (-.04) | **.42** (.37**)** | **.23**** |
| State anxiety | .12 (.11) | .12 (-.08) | **.28** (.39**)** | **.22*** |
| Trait anxiety | .12 (.19) | .09 (-.08) | **.36**** (.24) | **.24*** |
| Depression | **.27** (.36**)** | .17 (-.13) | **.31**** (.16) | **.35**** |
| Alexithymia | **.43** (.28*)** | .23 (.01) | **.33**** (.18) | **.48**** |
| **Impulsivity** | | | | |
| *BIS-11 total* | **.23*** (.19) | .10 (.12) | **.24*** (.24) | **.28**** |
| Nonplanning impulsivity | .04 (.10) | -.02 (-.18) | .16 (.16) | .08 |
| Motor impulsivity | **.27*** (.18) | .13 (-.09) | .12 (.18) | **.27*** |
| Attentional impulsivity | **.27*** (.18) | .16 (.02) | **.31**** (.24) | **.35**** |
| *SSS-V total* | **.21*** (.26) | .13 (-.11) | .03 (.02) | .19 |
| Disinhibition | **.37** (.44**)** | **.24*** (-.06) | .11 (.02) | **.36**** |
| Boredom susceptibility | **.41* (.35*)** | **.31**** (.05) | .16 (.16) | **.43**** |
| Thrill and adventure seeking | -.05 (-.05) | -.04 (-.03) | -.11 (-.03) | -.09 |
| Experience seeking | -.12 (.04) | -.13 (-.25) | -.04 (-.04) | -.13 |
| *UPPS total* | .13 (.19) | -.01 -.07) | **.25* (.31*)** | .18 |
| Negative urgency | .14 (.13) | .09 (-.06) | **.46** (.55**)** | **.29**** |
| Positive urgency | **.37** (.28*)** | .20 (.02) | .25 (.24) | **.40**** |
| Premeditation (lack of) | -.01 (.02) | -.07 (-.09) | .01 (.04) | -.01 |
| Perseverance (lack of) | .16 (.18) | .01 (-.11) | .19 (.12) | .18 |
| Sensation Seeking | .06 (.01) | .03 (-.01) | -.01 (.04) | .05 |
| *WURS / ADHD total* | .21 (.03) | .09 (-.04) | .37 **(.44**)** | **.30**** |
| **Demographics** | | | | |
| Age | **-.22*** (.-.27) | -.20 (-.15) | -.12 (-.09) | **-.25*** |
| IQ (Raven matrices) | -.19 (-.07) | -.20 (-.11) | -.02 (.12) | -.19 |
| Years education | -.13 (-.17) | -.10 (.06) | -.01 (.12) | -.12 |
| Gender | **-.22*** (-.10) | **-.25*** (-.12) | .18 (.09) | -.17 |

*Note*: Zero-order correlations are outside the parentheses, partial correlations are inside the parentheses, values in bold are significant: ** at the level of 0.01 (2-tailed), * at the level of 0.05 (2-tailed).

**Table S3.** Convergent and discriminant validity: Zero-order and partial correlations between LSRP scales and criterion variables in polysubstance-dependent individuals.

| **Criterion Variables** | **Egocentric** | **Callous** | **Antisocial** | **Total** |
| --- | --- | --- | --- | --- |
| **Antisocial behavior** | | | | |
| Conduct disorder | .12 (.05) | .15 (.09) | .15 (.10) | .17 |
| Antisocial personality disorder | .11 (-.02) | **.28** (.23**)** | **.20**** (.14) | **.23**** |
| **Aggression** | | | | |
| **AQ total** | **.34**** (.17) | **.22*** (-.02) | **.53** (.48**)** | **.49**** |
| Physical aggression | **.29**** (.13) | **.28**** (.16) | **.40** (.37**)** | **.43**** |
| Verbal aggression | **.27**** (.13) | .15 (-.07) | **.37**** (.25) | **.36**** |
| Anger | **.21*** (.06) | .16 (-.05) | **.53** (.54**)** | **.38**** |
| Hostility | **.32**** (.19) | .08 (-.13) | **.29**** (.19) | **.35**** |
| **Internalizing psychopathology** | | | | |
| Anxiety sensitivity | **.24**** (.19) | .03 (-.16) | **.29**** (.21) | **.26*** |
| State anxiety | .15 (.01) | .06 (-.04) | **.31** (.39**)** | **.24*** |
| Trait anxiety | .17 (-.07) | **.27**** (.16) | **.46** (.53**)** | **.36*** |
| Depression | **.26**** (.05) | **.18*** (.13) | **.39** (.35**)** | **.37*** |
| Alexithymia | .19 (.06) | .18 (.08) | **.34** (.29*)** | **.29**** |
| **Impulsivity** | | | | |
| *BIS-11 total* | **.24**** (.02) | .12 (-.08) | **.58** (.56**)** | **.41**** |
| Nonplanning impulsivity | .14 (-.13) | .11 (-.06) | **.45** (.47**)** | **.29**** |
| Motor impulsivity | **.28**** (.12) | .08 (-.05) | **.52** (.49**)** | **.40**** |
| Attentional impulsivity | **.18*** (.09) | .11 (-.09) | **.49** (.48**)** | **.34**** |
| *SSS-V total* | **.21*** (.12) | .05 (.02) | **.23*** (.24) | **.25**** |
| Disinhibition | **.28**** (.19) | .11 (.08) | **.18*** (.18) | **.31**** |
| Boredom susceptibility | **.23*** (.19) | .06 (-.08) | **.26** (.32*)** | **.27**** |
| Thrill and adventure seeking | .10 (.05) | .06 (.10) | .12 (.06) | .13 |
| Experience seeking | -.07 (-.15) | -.13 (-.10) | .05 (.14) | -.07 |
| *UPPS total* | .17 (.08) | **.26*** (.11) | **.51** (.55**)** | **.39**** |
| Negative urgency | .16 (-.12) | **.26*** (.17**)** | **.59** (.55**)** | **.40**** |
| Positive urgency | **.31*** (.16) | **.26*** (.12) | **.43** (.36**)** | **.43**** |
| Premeditation (lack of) | .14 (-.12) | .13 (.05) | **.41** (.44**)** | **.29**** |
| Perseverance (lack of) | .15 (-.09) | .09 (-.13) | **.42** (.50**)** | **.29**** |
| Sensation Seeking | **.26**** (.24) | .13 (.08) | **.21*** (.04) | **.29**** |
| *WURS / ADHD total* | -.04 **(-.30*)** | .00 (-.15) | **.39** (.42**)** | .11 |
| **Demographics** | | | | |
| Age | -.06 (-.18) | .02 (.00) | .11 (.03) | .01 |
| IQ (Raven matrices) | **-.25**** (-.01) | -.12 (-.16) | -.08 (.02) | **-.23*** |
| Years education | -.07 (.05) | -.08 (-.14) | -.06 (-.09) | -.09 |
| Gender | **-.22*** (-.18) | -.11 (-.02) | -.12 (-.06) | **-.22*** |

*Note*: Zero-order correlations are outside the parentheses, partial correlations are inside the parentheses, values in bold are significant: ** at the level of 0.01 (2-tailed), * at the level of 0.05 (2-tailed).

**Table S4.** Convergent and discriminant validity: Zero-order and partial correlations between LSRP scales and criterion variables in the control group.

| **Criterion Variables** | **Egocentric** | **Callous** | **Antisocial** | **Total** |
| --- | --- | --- | --- | --- |
| **Antisocial behavior** | | | | |
| Conduct disorder | **.17** (.12*)** | .08 (.04) | **.13**** (.08) | **.19**** |
| Antisocial personality disorder | **.22** (.14*)** | **.22** (.18**)** | **.15**** (.10) | **.28**** |
| **Aggression** | | | | |
| **AQ total** | **.30**** (.03) | **.13*** (.08) | **.60** (.60**)** | **.48**** |
| Physical aggression | **.29**** (.09) | **.14*** (.01) | **.29** (.29**)** | **.36**** |
| Verbal aggression | .06 (-.06) | .09 (.07) | **.28** (.25**)** | **.18**** |
| Anger | **.15*** (-.10) | .02 (-.02) | **.54** (.57**)** | **.31**** |
| Hostility | **.36** (.20**)** | **.13* (.16*)** | **.49** (.46**)** | **.48**** |
| **Internalizing psychopathology** | | | | |
| Anxiety sensitivity | **.13* (.14*)** | .04 (.03) | **.27** (.28**)** | **.21**** |
| State anxiety | .10 (.02) | .09 (.04) | **.33** (.31**)** | **.23**** |
| Trait anxiety | .07 (-.09) | .02 (-.02) | **.52** (.49**)** | **.25**** |
| Depression | .09 (-.04) | .07 (.07) | **.40** (.37**)** | **.24**** |
| Alexithymia | **.29** (.17*)** | .12 (.08) | **.36** (.31**)** | **.38**** |
| **Impulsivity** | | | | |
| *BIS-11 total* | **.12*** (-.08) | .04 (-.03) | **.45** (.45**)** | **.27**** |
| Nonplanning impulsivity | .07 (-.07) | .06 (.-.02) | **.34** (.34**)** | **.19**** |
| Motor impulsivity | **.13*** (-.02) | -.01 (-.04) | **.33** (.33**)** | **.21**** |
| Attentional impulsivity | .11 (-.11) | .05 (.01) | **.45** (.45**)** | **.26**** |
| *SSS-V total* | **.17** (.08)** | .00 (-.11) | .09 (.05) | **.15**** |
| Disinhibition | **.30** (.17*)** | .08 (-.04) | **.13*** (.08) | **.28**** |
| Boredom susceptibility | .11 (.03) | .06 (-.01) | .06 (-.02) | **.15*** |
| Thrill and adventure seeking | **.12* (.09)** | -.03 (-.12) | .07 (.05) | .09 |
| Experience seeking | -.05 (-.09) | -.09 (-.11) | .01 (.02) | -.06 |
| *UPPS total* | **.12* (.02)** | .09 (.04) | **.48** (.49**)** | **.29**** |
| Negative urgency | **.15* (-.08)** | -.01 (-.08) | **.58** (.57**)** | **.32**** |
| Positive urgency | **.23* (.10)** | .08 (.06) | **.48** (.45**)** | **.38**** |
| Premeditation (lack of) | .01 (-.11) | **.16* (.16*)** | **.18** (.14*)** | **.12*** |
| Perseverance (lack of) | -.02 (-.13) | .08 (.07) | **.33** (.31**)** | **.13*** |
| Sensation Seeking | **.31* (.20**)** | .06 (-.06) | **.17**** (.12) | **.29**** |
| *WURS / ADHD total* | .08 (-.06) | -.04 (-.05) | **.42** (.34**)** | **.21**** |
| **Demographics** | | | | |
| Age | **-.18* (-.14*)** | -.09 (-.05) | -.02 (.04) | **-.16**** |
| IQ (Raven matrices) | **-.13* (-.02)** | **-.28** (-.36**)** | -.11 **(-.16*)** | **-.22**** |
| Years education | **-.28** (-.29**)** | **-.17** (-.14*)** | -.05 (-.03) | **-.26**** |
| Gender | **-.28** (-.29**)** | **-.19** (-.14*)** | **.13* (.19*)** | **-.20**** |

*Note*: Zero-order correlations are outside the parentheses, partial correlations are inside the parentheses, values in bold are significant: ** at the level of 0.01 (2-tailed), * at the level of 0.05 (2-tailed).
